# What Is Your Diagnosis? Lymph Node Aspirates From a Dog

**DOI:** 10.1111/vcp.70061

**Published:** 2025-11-03

**Authors:** Gillian Herbert, Kristina Meichner, Emily Rout, Troy Myers, Mackenzie Long

**Affiliations:** ^1^ Department of Pathology, College of Veterinary Medicine University of Georgia Athens Georgia USA; ^2^ Clinical Hematopathology Laboratory Colorado State University Fort Collins Colorado USA

**Keywords:** canine, composite lymphoma, flow cytometry, PARR, T‐zone lymphoma

## 1 Case Presentation

A 10‐year‐old intact male Cavalier King Charles Spaniel presented to his primary veterinarian for mild generalized peripheral lymphadenomegaly first noticed by his owner the previous week. Previous medical history included grade IV dental disease and presumptive mitral valvular dysplasia, but he was otherwise free of clinical signs at the time of presentation. Pertinent CBC findings revealed a mild normocytic, normochromic anemia (hematocrit 37.8%; reference interval [RI]: 38.3%–56.5%), an eosinopenia (0.028 K/μL; RI 0.07–1.49 K/μL), and a moderate thrombocytopenia (75 K/μL; RI 143–448 K/μL). Lymphocytes were within the reference interval (2.918 K/μL; RI 1.06–4.95 K/μL). The thrombocytopenia was confirmed on manual blood smear review; however, it consisted of large platelets, and platelet mass was estimated to be adequate. The biochemistry profile was unremarkable except for mildly increased alkaline phosphatase activity (167 U/L; RI 5–160 U/L). Serology results for *Ehrlichia canis* , *Ehrlichia ewingii* , *Borrelia burgdorferi* , *Anaplasma phagocytophilum* , *Anaplasma platys* , *and Dirofilaria immitis* (SNAP 4Dx Plus Test, IDEXX Laboratories) were negative. Fine‐needle aspirate biopsy samples from the enlarged prescapular and popliteal lymph nodes were submitted to the University of Georgia, Athens Veterinary Diagnostic Laboratory for cytologic evaluation (Figure 1).

**FIGURE 1:**
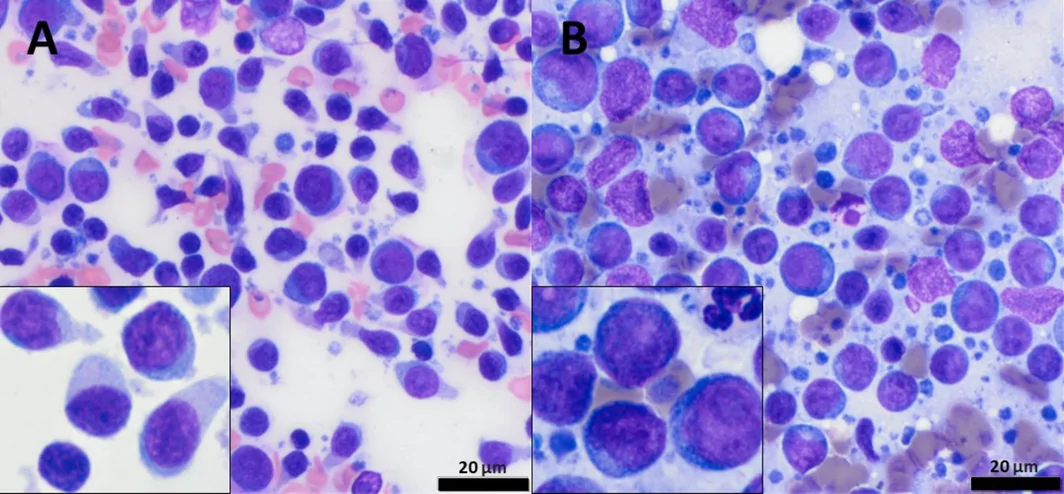
Photomicrograph of the fine‐needle aspirate biopsy cytology from the (A) prescapular and (B) popliteal lymph nodes of a dog with peripheral lymphadenomegaly; Modified Wright stain, 500× magnification (insets 1000× magnification).

### 1.1 Cytologic Interpretation

Probable lymphoma concerning for both T‐zone and large cell lymphoma.

## 2 Additional Results

Cytology of the prescapular lymph node (Figure 2A) consisted predominantly of small to intermediate lymphocytes (arrows) with a moderate number of erythrocytes and few free cytoplasmic fragments. These lymphocytes contained an asymmetric expansion of pale to lightly basophilic cytoplasm (pseudopods). The round to oval eccentric nucleus contained fine chromatin and indistinct nucleoli. Rare large lymphocytes (arrow heads) with scant, deeply basophilic cytoplasm and a perinuclear clear zone were observed. The round eccentric nucleus contained fine chromatin and multiple variably sized, distinct nucleoli. Cytology of the popliteal lymph node (Figure 2B) revealed an expansion of intermediate to large lymphocytes (arrowheads) with a morphology comparable to that of the large lymphocytes reported in the prescapular lymph node. The background contained mild blood contamination and numerous free cytoplasmic fragments. Occasional bizarre mitotic figures were seen. These findings were interpreted as concerning for both T‐zone and large cell lymphoma.

**FIGURE 2:**
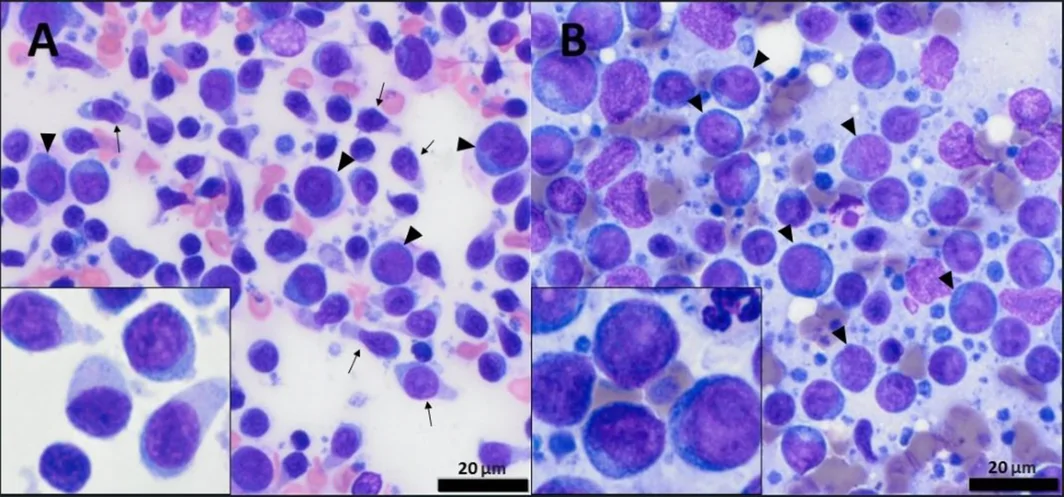
Photomicrograph of the fine‐needle aspirate biopsy cytology from the prescapular (A) and popliteal (B) lymph nodes of a dog with peripheral lymphadenomegaly. (A) The prescapular lymph node contains a predominant population of small to intermediate lymphocytes that have an asymmetrical cytoplasmic extension (arrows), interpreted as T‐zone cells. A smaller population of large lymphocytes (arrowhead) is also present. (B) The popliteal lymph node is composed predominantly of large lymphocytes (arrowhead). Modified Wright stain; 500× magnification (insets 1000× magnification).

Flow cytometry was performed on both lymph node fine‐needle aspirate biopsy samples through the Clinical Flow Cytometry Service at the University of Georgia College of Veterinary Medicine. The prescapular lymph node (Figure 3A) was interpreted as a probable emerging B‐cell lymphoma (BCL) based on the expansion of large B‐cells (~50%) and suspected concurrent T‐zone lymphoma (TZL) based on the population of T‐zone cells (~40%) with phenotypes as previously described. The popliteal lymph node (Figure 3B) was consistent with BCL based on the expansion (~90%) of large CD21^+^CD25^+^MCHII^+(bright)^CD45^+^ B‐cells. A subset (~4%) of small to intermediate T‐zone cells (CD45^−^CD3^+^CD5^+^CD25^+^MHC‐II^+^CD4^+/‐^CD8^−^ CD21^dim^) was also identified.

**FIGURE 3:**
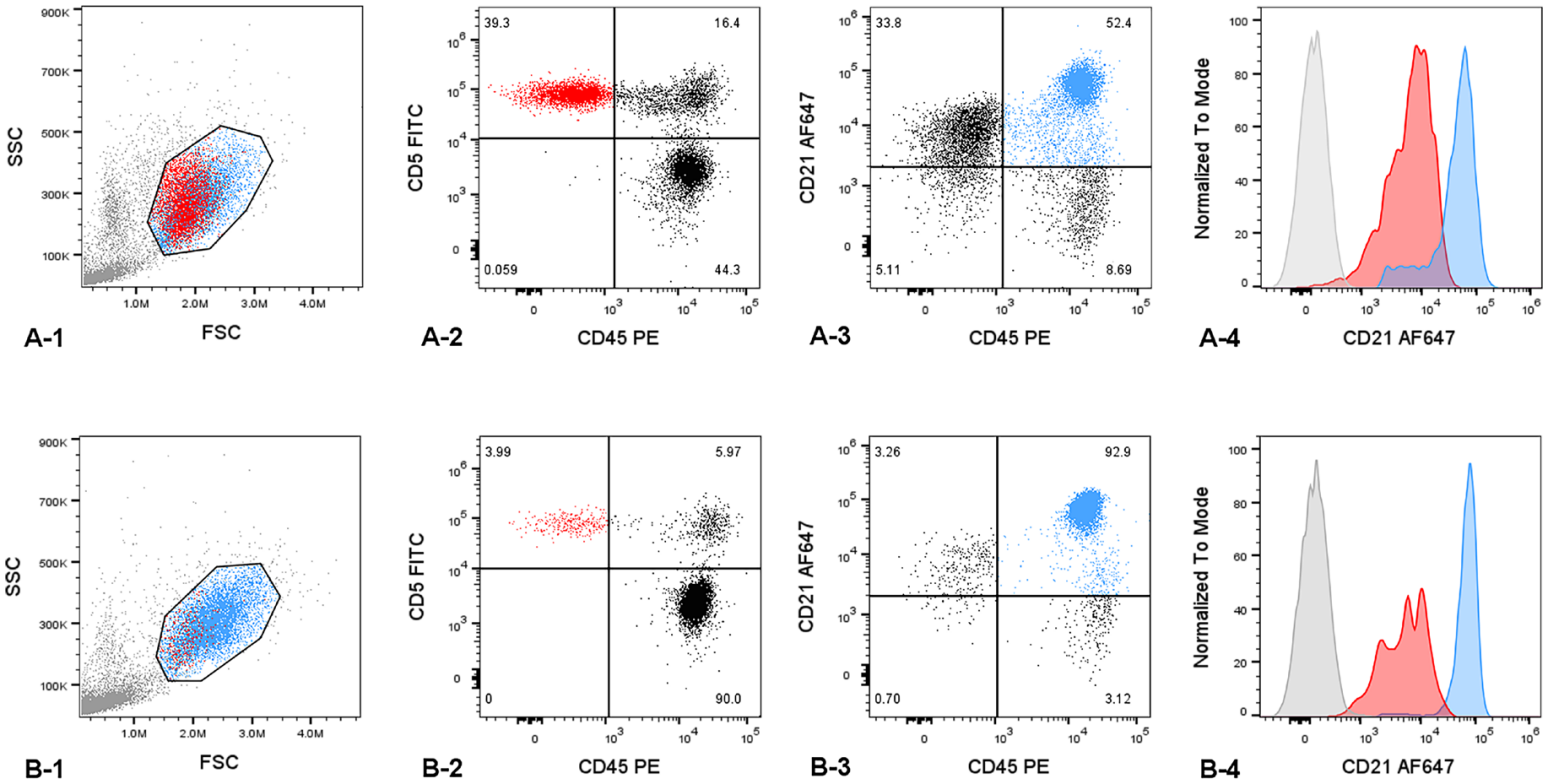
Immunophenotyping via flow cytometry of prescapular (A) and popliteal (B) lymph node fine‐needle aspirate biopsy samples from a dog with peripheral lymphadenomegaly. General gating on all cells in the sample based on forward (FSC) vs. side scatter (SSC) (A‐1, B‐1) revealed two distinct cell types. The first population is comprised of CD45^−^CD5^+^CD21^dim^ T‐zone cells (red color), composing ~39.3% of cells in the prescapular (A‐2) and ~3.99% of cells in the popliteal lymph node aspirate (B‐2), respectively. The second population consists of large CD45^+^CD21^+^ B‐cells (blue color) composing ~52.4% of cells in the prescapular (A‐3) and ~92.9% of cells in the popliteal lymph node aspirate (B‐3), respectively. Histogram of CD21 staining intensity for both lymph node aspirates (A‐4/B‐4) showing higher CD21 intensity on the CD45^+^CD21^+^ B cells (blue) compared to the CD45^−^CD5^+^ T zone cells (red). Control (isotype) depicted in gray. Counts on the *Y*‐axis were normalized to the number of cells. Analysis was performed on singlets and after exclusion of 7‐AAD^+^ dead cells (not shown) for cells within one tube with the following antibody (clone) combination: CD4 (YKIX302.9), CD5 (YKIX322.3), CD8alpha (YCATE55.9), CD21 (CA2.1D6).

Colorado State University's College of Veterinary Medicine and Biomedical Sciences Clinical Hematopathology Laboratory conducted PCR for antigen receptor rearrangements (PARR) on the combined aspirates as previously described [1]. This revealed clonal rearrangements of both the T‐cell receptor and immunoglobulin genes, supporting the presence of concurrent T‐cell and B‐cell lymphoma, respectively (Figure 4).

**FIGURE 4:**
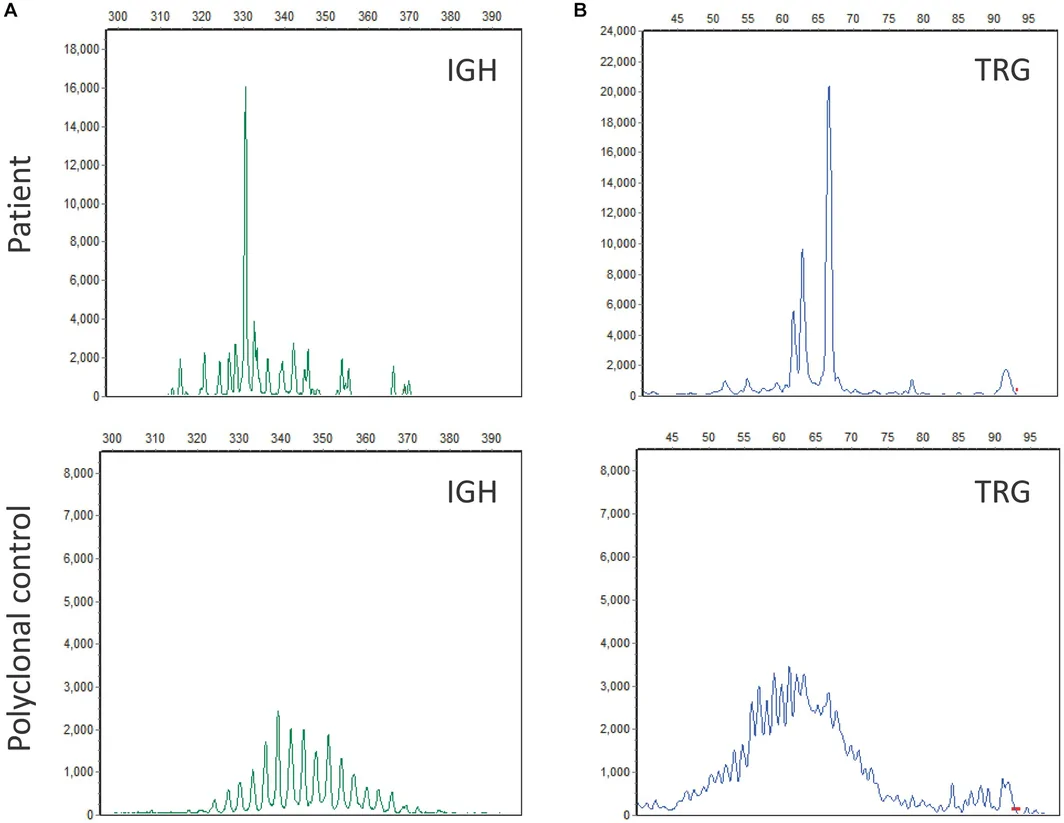
PCR for antigen receptor rearrangements (PARR) results from a combined lymph node aspirate of a dog with peripheral lymphadenomegaly. Immunoglobulin heavy chain (A) and T cell receptor gamma (B) amplification of DNA from the patient sample. Results demonstrate a clonal IGH rearrangement and clonal TRG rearrangement, defined as a tall, narrow peak that exceeds 5000 in amplitude and is 3× the height of the peaks forming the polyclonal background. Insets: Polyclonal negative control from a normal dog.

Chemotherapy was not pursued. The dog did well clinically for 3 months, during which time he received a short course of low‐dose prednisone for an episode of back pain. Treatment with immunosuppressive doses of prednisone was discussed but not elected due to pre‐existing heart disease. He was ultimately euthanized after an episode of vomiting and difficulty walking. A post‐mortem examination was not performed.

## 3 Discussion

The present case describes suspected composite nodal BCL and TZL in a dog. Composite lymphoma refers to the coexistence of two lineage‐distinct lymphoma malignancies in one or multiple anatomic sites of one patient [2]. Several reports describe suspected composite lymphoma involving TZL in dogs [3, 4, 5], and a retrospective case series of 51 dogs with TZL found that approximately 12% developed second malignancies [6]. The pathogenesis of composite lymphoma in dogs is poorly understood. In this case, the referring veterinarian reported a history of T‐zone lymphoma in a littermate, prompting speculation that a shared genetic predisposition may have contributed to the development of T‐zone lymphoma in this dog.

Differentiating de novo tumor development from malignant transformation (e.g., Richter syndrome) can be challenging and cannot be reliably accomplished using standard clonality assays alone. In the present case, composite lymphoma was highly suspected based on distinct cytomorphology, immunophenotypes, and the presence of two different clonal rearrangements within the lymphoid population. However, lineage distinction based on PARR has not been validated in the context of Richter syndrome or composite lymphoma, particularly given the potential for cross‐lineage rearrangements in some T‐zone lymphomas [7]. Therefore, next‐generation sequencing to quantify and characterize antigen receptor gene rearrangements is preferable but was not performed in this case.

Composite lymphoma presents a unique diagnostic challenge. From a cytologic perspective, the presence of two morphologically distinct lymphoid populations may prompt a variety of other considerations, including lymphoid hyperplasia, early or partial effacement of a lymph node by lymphoma, aspiration of a germinal center, or T‐cell‐rich B‐cell lymphoma. This highlights the importance of additional diagnostic investigation to achieve an accurate diagnosis. Since approximately 60% of dogs with TZL present with lymphocytosis [6, 7], the presence of concurrent lymphocytosis characterized by small to intermediate lymphocytes may increase the index of suspicion for multiple lymphoid malignancies, though it was not a feature of this case.

In humans, the clinical course for composite lymphoma is thought to be dictated by the more malignant entity [2]. In veterinary species with composite lymphoma, monitoring disease progression using response evaluation criteria in solid tumors is challenging [4]. Several reports indicate persistent lymphadenomegaly in dogs with concurrent TZL and DLBCL due to TZL's chemotherapy resistance [3, 4, 5]. Unfortunately, no additional information regarding response to treatment or prognosis can be elucidated from this case as chemotherapy was not pursued, and the cause of clinical decline was undetermined.

In addition to the lack of follow‐up, there are several limitations in this case. The fact that PARR was inadvertently run on a combined sample rather than on each affected lymph node limited direct correlation with cytology and flow cytometry findings. Additionally, tissue samples were unavailable for histopathology, which is a valuable tool in suspected cases of composite lymphoma to confirm the architectural separation of the two neoplasms within the lymph node. In summary, this report describes a case of suspected composite nodal TZL and BCL occurring within the same lymph nodes. Composite lymphomas pose diagnostic challenges and have implications for monitoring disease progression, underscoring the importance of diagnostic tests beyond cytology to accurately classify lymphomas in dogs.

## Conflicts of Interest

The authors declare no conflicts of interest.
